# Identification of a novel *GRIN2D* variant in a neonate with intractable epileptic encephalopathy-a case report

**DOI:** 10.1186/s12887-020-02462-6

**Published:** 2021-01-04

**Authors:** Jiancheng Jiao, Li Li, Min Sun, Junchen Fang, Lingzhi Meng, Yudong Zhang, Chao Jia, Li Ma

**Affiliations:** Neonatology Department, Children’s Hospital of Hebei Province, 133 Jianhua South Street, 050031 Shijiazhuang, Hebei Province China

**Keywords:** case report, NMDA receptors, *GRIN2D* variants, de novo mutation, epileptic encephalopathy, anti-epileptic medications

## Abstract

**Background:**

N-methyl-D-aspartate (NMDA) receptors are ligand-gated ion channels that mediate excitatory synaptic transmission in the central nervous system. The functional NMDA receptors are heterotetramers consisting mainly of two GluN1 and two GluN2 subunits. GluN2 is encoded by the *GRIN2D* gene. A few case series have shown that *GRIN2D* variants are linked to developmental and epileptic encephalopathy. In this article, we report a novel *GRIN2D* variant, namely c.2021C > A (p.T674K) in a neonate with intractable epileptic encephalopathy.

**Case presentation:**

A 12-day-old boy who had stiffness of the lower and upper extremities since birth was transferred from a local hospital to our department. On admission, the patient presented with head tilting backwards, staring, apnea and hypertonia of limbs. Video electroencephalogram showed continuous, generalized or multi-focal spike-wave and spike-and-slow wave discharges and hypsarrhythmia. A treatment regimen composed of phenobarbital, midazolam, levetiracetam and clonazepam was administered, which however led to only partial control of the seizure. Whole-exome sequencing identified c.2021C > A (p.T674K) in *GRIN2D* in the patient while such a mutation was not detected in the parents. The patient was hospitalized for 1 month and died of sudden cardio-respiratory arrest 2 weeks after discharge.

**Conclusions:**

A novel variant of *GRIN2D* was identified in a neonate with epileptic encephalopathy. Epilepsy associated with this *GRIN2D* mutation is refractory to conventional anti-epileptic medications.

## Background

N-methyl-D-aspartate (NMDA) receptors are ligand-gated ion channels that are expressed throughout the central nervous system and mediate excitatory synaptic transmission [1]. To date, seven subunits of NMDA receptors, i.e., the GluN1 subunit, four distinct GluN2 subunits (GluN2A, GluN2B, GluN2C and GluN2D encoded by four different genes), and two GluN3 subunits (GluN3A and GluN3B) have been identified [2]. The functional NMDA receptors are heterotetramers consisting mainly of two GluN1 and two GluN2 subunits [3]. GluN2 is encoded by the *GRIN2D* gene [1]. A few case series have shown that *GRIN2D* variants are linked to developmental and epileptic encephalopathy [4–6]. In this study, we describe a novel de novo mutation in *GRIN2D* in a neonate with intractable epileptic encephalopathy.

## Case presentation

A 12-day-old boy presenting with stiffness in the lower and upper extremities since birth was transferred from a local hospital to our department. He was born at full-term without asphyxia to a G2P1 mother, with a birth weight of 3,600 g and an Apgar score of 9 and 10 at 1 and 5 minutes, respectively. Placenta, umbilical cord and amniotic fluid appeared normal. The parents were unrelated and healthy, and had no family history of epilepsy. On admission, the patient presented with head tilting backwards, staring, apnea and hypertonia in upper and lower limbs. He had a modified Ashworth scale score of 4 according to following findings: clenched fists with fingers fully flexed into the palm; flexed upper limbs to the chest; tonic extended lower limbs with flexed toes; and absence of sucking reflex, rooting reflex and Moro reflex. His temperature was 37 °C, heart rate 100/min, blood pressure 86/50 mmHg and SpO2 60%.

**Fig. 1:**
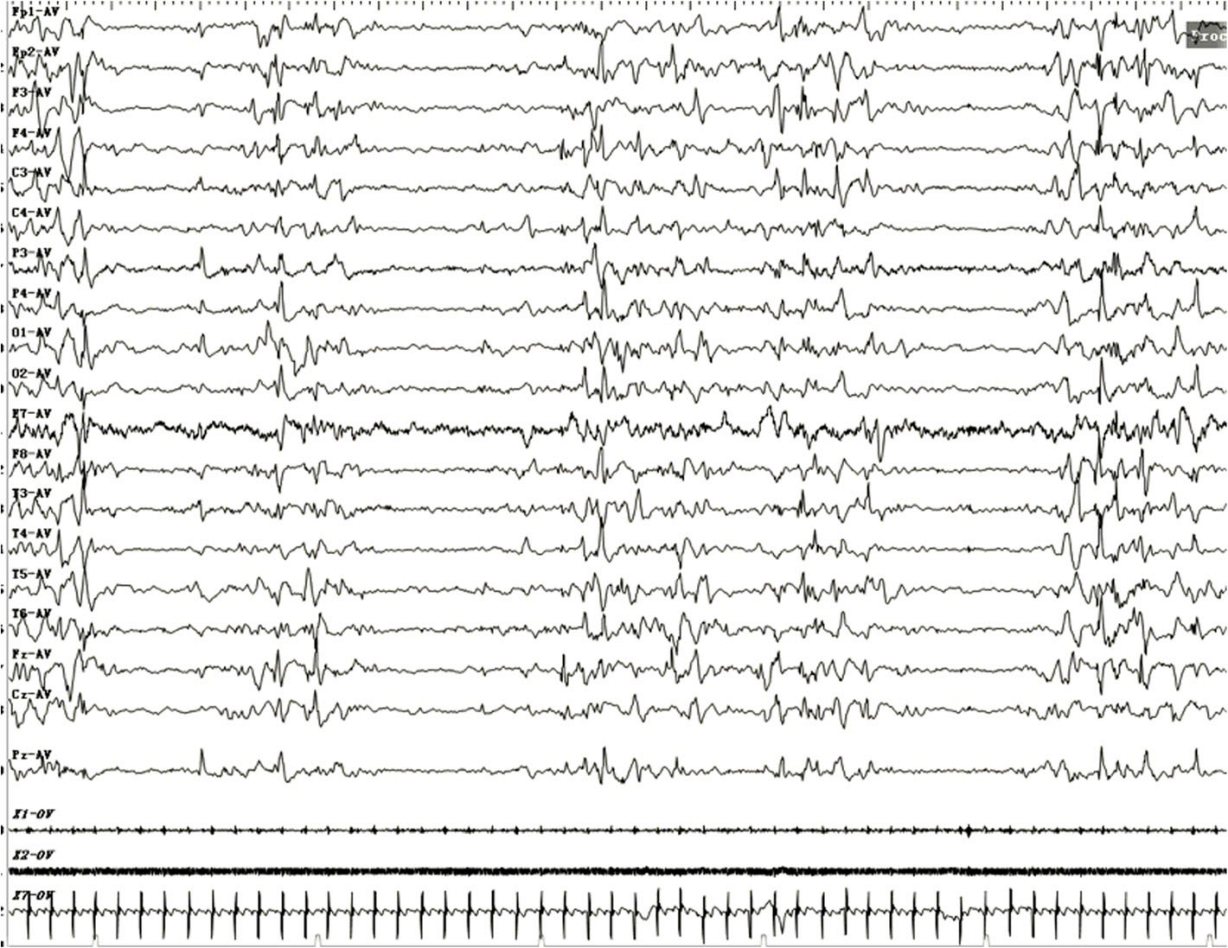
Electroencephalogram of the patient. Intermittent or continuous discharges (hypsarrhythmia) of generalized or multi-focal spike-waves and spike-and-slow waves were recorded by electroencephalography

Blood, cerebrospinal fluid (CSF), urine and stool testing results were unremarkable. No growth was reported in blood and CSF culture. Cardiac enzyme profiling, electrolyte analyses, blood amino acid and acylcarnitine spectrum analyses for inherited metabolic diseases, and comprehensive panel of urine organic acids were unrevealing. Thyroid, liver and renal functions were normal. Cytomegalovirus and herpes virus, and their respective antibodies were negative as determined by PCR and ELISA, respectively. Chest radiography, electrocardiography, echocardiography and head MRI did not identify abnormalities. The automated auditory brainstem response was normal. Video electroencephalogram (EEG) was recorded and showed intermittent or continuous discharges (hypsarrhythmia) of generalized or multi-focal spike-waves and spike-and-slow waves (Fig. 1), in line with epileptic encephalopathy.

**Fig. 2:**
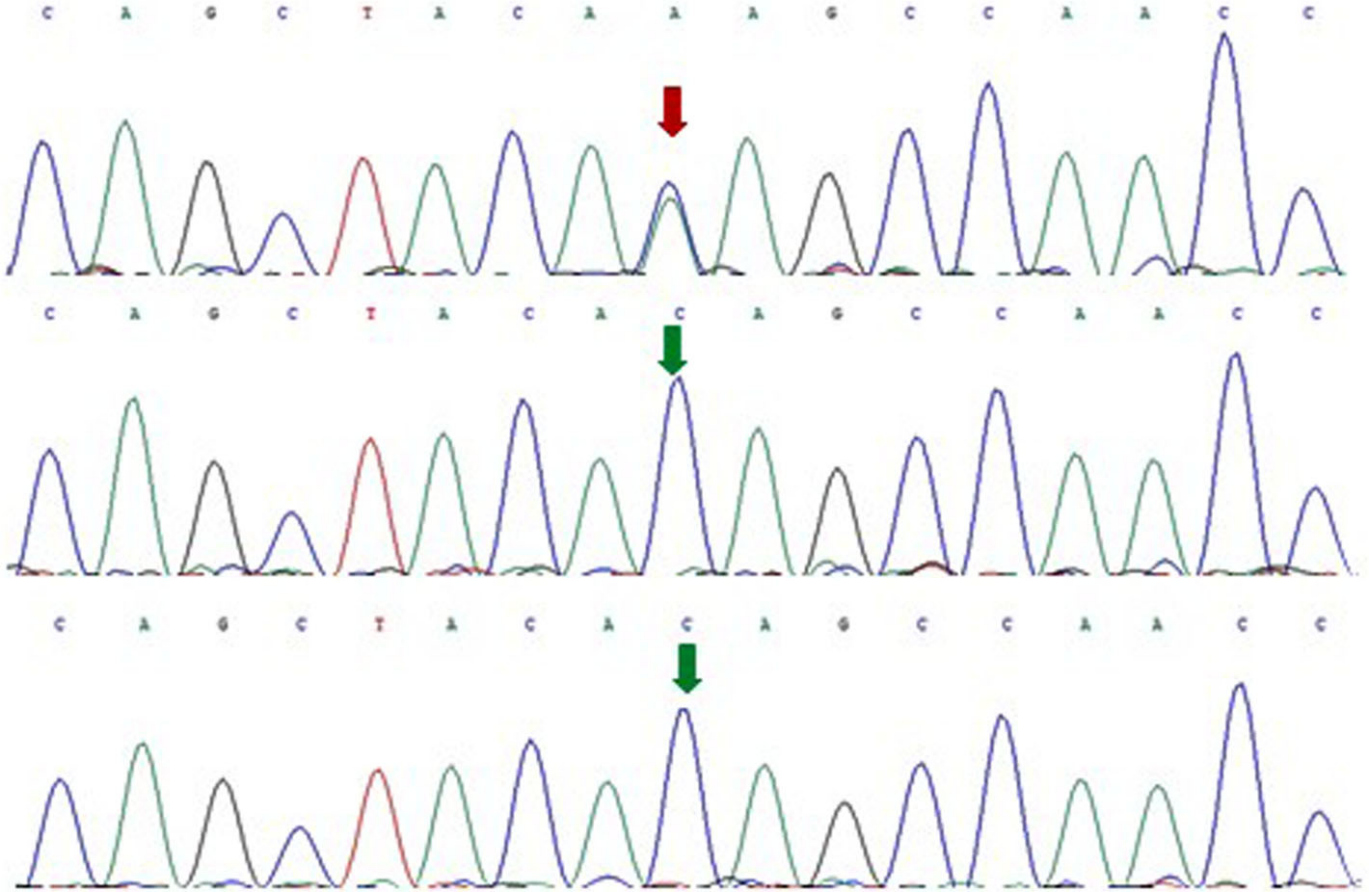
Histogram of whole-exome sequencing (proband: top, father: middle, and mother: bottom). A heterozygous missense mutation, i.e., c.2021C > A (p. Thr674Lys) in *GRIN2D* was detected in the patient but not in the parents

The neonate was intubated and ventilated immediately after admission, and nasogastric feeding instituted. Phenobarbital, diazepam and vitamin B6 were initiated, which however did not show effect in seizure control (over 10 episodes/day). Diazepam was discontinued and a bolus intravenous injection of midazolam (0.15 mg/kg) was given followed by slower maintenance (0.2 mg/kg/h), which led to seizure reduction to ~ 10 episodes/day. On day 5 of admission, levetiracetam and clonazepam were added to the regimen, which significantly reduced seizure severity and frequency (5–6 episodes/day). Midazolam was tapered off in 2 weeks. Eighteen days after admission, the patient was extubated. Consent to taking a genetic test was obtained from the parents, and blood was drawn for whole-exome sequencing which was performed by Beijing Fulgent Technologies Inc. (Beijing, China). A heterozygous missense mutation, i.e., c.2021C > A (p. Thr674Lys) in *GRIN2D* in the patient was detected while such a mutation was not found in the parents (Fig. 2). The patient was discharged 1 month after hospitalization with continuous nasogastric administration of levetiracetam and clonazepam at home. Two weeks after discharge, the patient died of sudden cardio-respiratory arrest.

## Discussion and Conclusions

To date, ten *GRIN2D* variants found in a total of 13 patients with developmental and epileptic encephalopathy have been described in the literature, which include Val667Ile, Met681Ile, Ser694Arg, Asp449Asn, Ser573Phe, Leu670Phe, Ala675Thr, Ala678Asp, Ser1271Leu and Arg1313Trp [4–6]. Clinical features, neurological context, treatment and outcomes of these patients have been summarized [4–6]. In this study, we identified a de novo missense mutation, i.e., Thr674Lys in *GRIN2D*, which is novel. According to the criteria for classifying pathogenic variants established by the American College of Medical Genetics and Genomics [7], the case we present here renders strong evidence of pathogenicity for this variant since (1) Thr674Lys was detected in the patient but not in the parents, (2) both maternity and paternity were confirmed, and (3) the patient had the disease without family history.

In the largest case series of 8 patients reported thus far, 7 received multiple anti-epileptic medications including clonazepam, levetiracetam, topiramate, vigabatrin and valproate, but only 2 completely responded to the treatment while 3 had no response and 2 achieved mild amelioration [4]. Li et al. studied 2 patients with Val667Ile and reported that both individuals’ seizures were refractory to conventional antiepileptic medications [6]. Tsuchida et al. revealed ineffectiveness of clobazam and levetiracetam in one patient, and pyridoxine and clobazam in another in a case series of 3 patients [5]. In the present study, the neonate was initially treated with phenobarbital and diazepam, which however did not show effect, and the seizure was eventually partially controlled by administration of multiple drugs, i.e., phenobarbital, midazolam, levetiracetam and clonazepam. These data suggest the majority of patients with *GRIN2D* mutations are refractory to multiple anti-epileptic medications.

Functional analysis of the mutation was not pursued in this study. XiangWei et al. and Li et al. have characterized several *GRIN2D* variants, and reported that Val667Ile, Leu670Phe, Ala675Thr and Ala678Asp had enhanced while Ser1271Leu and Arp1313Trp had reduced receptor activities [4, 6]. Of interest, based on the finding that Val667Ile is a gain-of-function mutation, Li et al. conducted a therapeutic trial using the NMDA receptor antagonist memantine to treat two patients with Val667Ile who were refractory to conventional antiepileptic medications, and reported mild to moderate improvement in seizure in both cases after memantine treatment [6]. In the future, functional testing of novel *GRIN2D* variants, which differentiates gain-of-function mutations from loss-of-function ones, may lead to more effective management of epilepsy by tailoring medical treatment to the individual characteristics of each variant, e.g., use of NMDA receptor antagonists for the management of epilepsy caused by *GRIN2D* gain-of-function mutations.

In conclusion, we identified a novel *GRIN2D* variant in a neonate with intractable epileptic encephalopathy. Our data together with previously reported findings suggest that epilepsy caused by *GRIN2D* mutations is predominantly refractory to conventional anti-epileptic therapies. Functional characterization of mutants might provide guidance for personalized medicine for epilepsy associated with *GRIN2D* mutations.

## Data Availability

Data related to the case are presented in this article.
